# Supplementary material for: Effect of safety and security equipment on patient and visitor violence towards nurses in multiple public hospitals of China during the COVID-19 pandemic: a retrospective, difference-in-difference analysis
Source: BMJ Open. 2024 Jan 31;14(1):e078598. doi: 10.1136/bmjopen-2023-078598 (PMC10831433; doi:10.1136/bmjopen-2023-078598)
Supplement: Author's manuscript [file bmjopen-2023-078598.draft_revisions.pdf]

BMJ Open

BMJ Open is committed to open peer review. As part of this commitment we make the peer review history of every article we publish publicly available.

When an article is published we post the peer reviewers' comments and the authors' responses online. We also post the versions of the paper that were used during peer review. These are the versions that the peer review comments apply to.

The versions of the paper that follow are the versions that were submitted during the peer review process. They are not the versions of record or the final published versions. They should not be cited or distributed as the published version of this manuscript.

BMJ Open is an open access journal and the full, final, typeset and author-corrected version of record of the manuscript is available on our site with no access controls, subscription charges or pay-per-view fees (http://bmjopen.bmj.com).

If you have any questions on BMJ Open's open peer review process please email info.bmjopen@bmj.com

BMJ Open

Effect of Safety and Security Equipment on Patient and Visitor Violence toward Nurses during the COVID-19 Pandemic

Journal:	*BMJ Open*
Manuscript ID	bmjopen-2023-078598
Article Type:	Original research
Date Submitted by the Author:	06-Aug-2023
Complete List of Authors:	Hu, Yanzhen; Beijing Jishuitan Hospital,<br>Huang, Ju; Chinese Academy of Medical Sciences and Peking Union Medical College, Institute of Medical Information<br>Xia, Jinghua; Beijing Jishuitan Hospital, Department of Nursing<br>Zhang, Cheng; Chinese Academy of Medical Sciences & Peking Union Medical College Institute of Medical Information<br>Zhao, Dan; Beijing Jishuitan Hospital, Department of Nursing<br>Lu, Xue-mei; Jishuitan Hospital and Fourth Medical College of Peking University, Department of Nursing
Keywords:	Nurses, COVID-19, China, Health & safety < HEALTH SERVICES ADMINISTRATION & MANAGEMENT

SCHOLARONE™
Manuscripts

I, the Submitting Author has the right to grant and does grant on behalf of all authors of the Work (as defined in the below author licence), an exclusive licence and/or a non-exclusive licence for contributions from authors who are: i) UK Crown employees; ii) where BMJ has agreed a CC-BY licence shall apply, and/or iii) in accordance with the terms applicable for US Federal Government officers or employees acting as part of their official duties; on a worldwide, perpetual, irrevocable, royalty-free basis to BMJ Publishing Group Ltd ("BMJ") its licensees and where the relevant Journal is co-owned by BMJ to the co-owners of the Journal, to publish the Work in this journal and any other BMJ products and to exploit all rights, as set out in our licence.

The Submitting Author accepts and understands that any supply made under these terms is made by BMJ to the Submitting Author unless you are acting as an employee on behalf of your employer or a postgraduate student of an affiliated institution which is paying any applicable article publishing charge ("APC") for Open Access articles. Where the Submitting Author wishes to make the Work available on an Open Access basis (and intends to pay the relevant APC), the terms of reuse of such Open Access shall be governed by a Creative Commons licence – details of these licences and which Creative Commons licence will apply to this Work are set out in our licence referred to above.

Other than as permitted in any relevant BMJ Author's Self Archiving Policies, I confirm this Work has not been accepted for publication elsewhere, is not being considered for publication elsewhere and does not duplicate material already published. I confirm all authors consent to publication of this Work and authorise the granting of this licence.

For peer review only

Effect of Safety and Security Equipment on Patient and Visitor Violence toward Nurses during the COVID-19 Pandemic

Yan-zhen Hu[1#], Ju Huang[2#], Jing-hua Xia[1], Cheng Zhang[2], Dan Zhao [1], Xue-mei Lu[1*]

[1]Department of Nursing, Beijing Jishuitan Hospital, Capital Medical University, Beijing, China
[2]Institute of Medical Information, Chinese Academy of Medical Sciences and Peking Union Medical College, Beijing, China

*** Correspondence:** Xue-mei Lu
jstlxm@163.com

[#] Yan-zhen Hu and Ju Huang are joint first authors.

Number of Words:3856

Number of Tables: 4

Abstract

Objectives This study aimed to analyze whether safety and security equipment decreased patient and visitor violence (PVV) toward nurses in the COVID-19 period and quantify to what extent safety and security equipment affects PVV.

Design Controlled before and after study and difference-in-difference analysis.

Setting A large Hospital Medical Group, consisting of three public tertiary teaching hospital, namely Xinjiekou Branch, Huilongguan Branch, and Xinlongze Branch of Beijing Jishuitan Hospital, located in the west and north parts of Beijing, China.

Participants Panel of nine department recruited using two-step sampling method, administered online surveys in 2021 and 2022. A total of 632 eligible nurses participated in the survey in 2021, 725 eligible nurses in 2022.

Measures We assessed impacts of the safety and security equipment on the PVV. The policy has been enacted in June 2020, and the corresponding measures was established after in mid-December 2020, and therefore we use a difference-in-differences design to investigate changes in nurses' PVV incidence. Departments are classified as either department-installed or non-installed and nurses are classified based on their department.

Results Within the treatment group, the incidence of physical PVV significantly decreased from 13.8% in 2020 to 2.0% in 2021. In the control group, the incidence of physical PVV increased from 0.6% in 2020 to 2.7% in 2021. The application of the safety and security equipment decreased the incidence of physical PVV by 13.93% (95%CI: -23.52% to -4.34%). In contrast, no effect was observed between the treatment and control groups for the incidence of psychological PVV (6.23%, 95%CI: -11.56% to 24.02%) and overall PVV (0.88, 95%CI: -20.90% to 22.66%).

Conclusion The safety and security equipment resulted in a reduction on the incidence of physical PVV. For hospital managers, longer-term strategies roadmap for PVV prevention measures are needed to create a more supportive work environment in employees.

Keywords safety and security equipment, nurses, workplace Violence, difference-in-difference, COVID-19

Introduction

Violence and harassment against health workers (HWs) have been increasing, especially as the world fights coronavirus disease 2019 (COVID-19), which has been ongoing for 3 years, putting HWs under tremendous stress in hospitals. [1,2] Workplace violence (WPV) is a serious threat to HWs; the Bureau of Labor Statistics notes that of 15,980 private industry workers who experienced trauma from nonfatal WPV, 69 percent worked in health care. [3] The International Labour Office (ILO) defines WPV as incidents in which staff are abused, threatened, or assaulted in their work, which is divided into physical and psychological violence. [4] The most prevalent type of WPV in healthcare is patient and visitor violence (PVV), which can lead to physical injury (e.g., beating, kicking, slapping, stabbing, shooting, pushing, biting, and pinching, among others) and psychological harm (e.g., verbal abuse, bullying/mobbing, harassment, and threats). [5]

Globally, there have been reports of frontline HWs, especially nurses, being attacked by perpetrators because of fear, panic, misplaced anger, and misinformation during the COVID-19 pandemic. [1] According to the International Committee of the Red Cross (ICRC), more than 600 cases of violence against HWs occurred in the first 6 months of the pandemic in the world, but cases have been reported are just only a tip of the iceberg. [6] Byon et al. [7] reported that 44.4% and 67.8% of nurses experienced physical and nonphysical violence, respectively, during a five-month period early in the COVID-19 pandemic in the United States. In China, many nursing staff, particularly experienced nurses, have worked on nasopharyngeal swab collection and other epidemy-related tasks, which may have resulted in nurses working long hours, insufficient rest, and constant overtime staff shortages. The shortage may aggravate the PVV. [8] In addition, anxiety, work pressure, and inadequate infection prevention and control (IPC) measures could have led to PVV during the early stage of COVID-19. [9]

With the improvement of IPC measures for frontline health workers in COVID-19, there are several countries which have developed and begun to implement coordinated strategies such as a “zero tolerance zone” for the prevention of WPV in the nurses. [10,11] The most basic element to protect against violence in the workplace is to have safe working conditions; thus, environmental controls are a key element of occupational violence and aggression prevention and management programs in healthcare worldwide, consisting of an increased presence of hospital security staff and equipment. [12] In China, the government approved the Medicine and Health Promotion Law, which took effect in June 2020 and included provisions to prevent PVV against medical workers. It emphasizes that no person or organization may threaten or endanger the personal safety of medical personnel or infringe upon their personal dignity. [6] Under this policy, safety and security measures at the organizational

For peer review only - http://bmjopen.bmj.com/site/about/guidelines.xhtml

and environmental levels were implemented for HWs by tertiary hospitals in mid-December 2020 in Beijing, including installing security cameras and alarm buttons, providing security staff with walkie-talkies, and increasing the numbers of metal detectors and security channels. [13]

The implementation of workplace hazard prevention and control measures associated with violence against HWs has been studied worldwide for years, and the measures can be divided into two types: training plans (for internal factors of PVV, perpetrator, or recipient characteristics) and organizational and environmental plans (for external factors, policies, and the workplace environment). [14] Currently, there are a few studies that have focused on external factors. Safety and security equipment as environmental prevention strategies attract little attention. Although research on the direct effects of safety and security equipment on the decrease in PVV during COVID-19 is limited, several positive research results were reported. A cross-sectional study by Henry Ford Hospital in Michigan evaluated installed metal detectors and found that they reduced violent crimes by 65% over 18 months. The metal detectors relieve the pressure on healthcare workers and decrease PVV. [15] Blando et al. [16] also found in a cross-sectional study that metal detectors can support weapon identification and removal, enhancing safety in healthcare facilities. Yet, owing to the limitations of the cross-sectional design in the existing research, the causal associations between safety and security equipment and PVV remain unknown.

Considering the scant evidence evaluating the efficacy of safety and security equipment on PVV prevention, particularly regarding nurses, we used panel data from 2020 to 2021, 2 years before and after the implementation of safety and security equipment, respectively, adopted a difference-in-differences (DID) design, to examine the effects of safety and security equipment on PVV against nurses. We provide unique and more objective findings in understanding the role of safety and security equipment in preventing PVV in China.

Methods

Study Design and data collection

We used data from a panel of departments located at two sites in the west and north of Beijing, within a large, public tertiary hospital group. The hospital that provides healthcare services across nationwide, with 1,600 beds, 3,509 employees and the yearly outpatient amount reaches 2.24 million. Baseline data were collected from January in 2021. In January 2022, we followed-up with respondents in selected departments who previously consented to participate in the panel. The inclusion criteria were full-time employees with qualification certificates and a willingness to answer the survey. The exclusion criterion was being on leave for more than a month in their department. The sample size was calculated using the double population proportion formula in PASS software based on the following assumptions: the prevalence of physical PVV from a previous study as 23% [17], 80% power to detect a significant difference of 0.05, an effect size of 0.1, and an allocation ratio of 1:3 between two groups. Finally, after accounting for a 10% non-response rate, the sample size of this study minimum yielded 517 respondents each year (120 for the treatment group and 397 for the comparison group). All surveys

For peer review only - http://bmjopen.bmj.com/site/about/guidelines.xhtml

were administered online by using Wenjuanxing Forms, which is the largest computer program for conducting online surveys in China to help researchers collect data without face-to-face meetings by sending online links to nurses through WeChat. According to the set rules, all items had to be completed before submission, and there were no missing values. Therefore, the Forms which has the function of checking the questionnaire automatically to ensure all questions were answered. [18,19] All participants in the survey were provided written information regarding the objective, context, and voluntary nature of their involvement in the study. The anonymity of all participants and the confidentiality of their responses were ensured. This study was approved by the Ethical Review Committee of the Chinese Academy of Medical Sciences (IMICAMS/8/22/HREC).

In 2021(pre-treatment), respondents were recruited using a two-step sampling method. First, the department-level samples were selected by setting up safety and security equipment, which covered all major types of wards involving nurses. The final sample comprised nine departments, and at least two wards were included for each department. Convenience sampling was performed to collect individual-level data from each department. The number of nurses in each selected department was determined and used as the sampling frame. The total sample size was allocated based on the sampling frame, resulting in 10–280 participants in each department. In 2022 (post-treatment), the same sampling method was used to select the study participants. Ultimately, nine departments were enrolled: three in the treatment group and six in the control group. A total of 632 eligible nurses participated in the survey in 2021, of whom 152 were from the treatment group and 480 were from the control group. In 2022, 725 eligible nurses answered the survey with 199 respondents from the treatment group and 526 from the control group.

Measures

The post-period for this analysis is defined as 2021. While the Medicine and Health Promotion Law was signed in June 2020, implementation was not instantaneous. The safety and security equipment were installed in the hospital in December 2020 and identified by the hospital security officer.

We evaluated the effect of safety and security equipment on the incidence of PVV among nurses in China. We ascertained that the data were broadly classified as either department-installed or non-installed safety and security equipment. The data for each installed department were further subclassified into pre-installed and installed period data. For each non-installed department, data were further subclassified into 'corresponding pre-installed period' and 'corresponding installed period' data. The outpatient department (OD), emergency department (ED), and technical services in the first department used safety and security equipment in January 2021 and were selected as the treatment group. Simultaneously, non-installed departments (the remaining six) were selected as the control group: general medicine, general surgery, operating room, intensive care, specialized unit, and management.

For peer review only - http://bmjopen.bmj.com/site/about/guidelines.xhtml

We measured several outcomes including physical PVV, psychological PVV and overall PVV. We used the structured Chinese Version-Revised of The Workplace Violence in the Health Sector Country Case Studies Research questionnaire. The English version was developed in 2003 by the International Council of Nurses (ICN), the World Health Organization (WHO), and Public Services International (PSI). [20] This questionnaire was revised based on the context of Chinese township hospitals and has been widely used in Chinese public hospitals. [21] The International Questionnaire-C-R includes the following four categories: demographic and workplace data; physical violence within the past 12 months; psychological workplace violence (verbal abuse, bullying/mobbing, sexual harassment, and racial harassment) within the past 12 months; and perception of effective strategies for PVV prevention. The reliability and validity of the questionnaire have been tested in studies involving experts from different areas of health in China. The Cronbach's alpha was 0.828. [22]

Finally, we measured individual-level controls, including age, marital status, job position, work experience, work in shift, work between 6PM and 7AM, interact with patients/clients and numbers of staff in the same work setting.

Statistical Analysis

We first present descriptive statistics of our sample, differences in PVV rates were compared using Pearson's Chi-squared test. Next, we assess the incidence changes of PVV in nurses that occurred pre-treatment versus post-treatment by using DID model. To obtain the significance level of the DID estimate, we adopted a regression model of the general form [23]:

$$y_{i,t}=\boldsymbol{\alpha}+\beta D_i+\gamma T_t+\delta I_{i,t}+\varepsilon_{i,t} \quad (1)$$

where the subscript i indicates department, t year, and $y_{i,t}$ denotes the outcome variable on the incidence of PVV varying across departments and years. D_i is the fixed effect of the control and treatment groups (1 for the department with complete safety and security equipment and 0 for the department without the equipment); T_t is the fixed effect of the pre- and post-treatment periods (1 for 2021 and 0 for 2020); and $I_{i,t}$ is a dummy equal to 1 for treatment individuals in the post-treatment period (otherwise, it is zero), that is,

$$I_{i,t}=D_i\times T_t \ (24,25) \quad (2)$$

All data analyses were conducted using Stata 16.0. The results were considered statistically significant at p-values<0.05.

Results

The sociological characteristics of the treatment and control groups in 2020 and 2021 are presented in Table 1. A total of 1357 nurses (632 in 2021 and 715 in 2022) were included. From 2021 to 2022, nearly 80% of participants were aged < 40 years old. More than 95% were female and nearly 60%

For peer review only - http://bmjopen.bmj.com/site/about/guidelines.xhtml

were married. Of the respondents, the majority (67.9% in 2021 and 65.8% in 2022) of respondents were senior nurses, and nearly 70% of respondents needed to work in shifts. More than 90% of respondents were female (95.1% in 2021 and 95.9% in 2022, respectively). On average, more than half of the respondents had junior titles (67.9% in 2021 and 65.8% in 2022, respectively). Regarding their work experience, more than 75% of all respondents had less than 15 years working experience. There were no statistical differences in the sociological characteristics in both the pre- and post-intervention periods between the treatment and control groups, indicating that the two groups were comparable (p>0.05).

Table 2 shows the proportions of PVV exposure in the treatment and control groups in 2020 and 2021. Within the treatment group, the incidence of physical PVV significantly decreased from 13.8% in 2020 to 2.0% in 2021 (χ^2=18.16, p<0.05) and psychological PVV decreased from 35.5% in 2020 to 28.6% in 2021 (χ^2=1.89, p>0.05). In the control group, the incidence of physical PVV increased from 0.6% in 2020 to 2.7% in 2021(χ^2=6.27, p<0.05), and psychological PVV decreased from 30.6% in 2020to 23.0% in 2021 (χ^2=7.46, p<0.05). These results support the effect of safety and security equipment on nurses' exposure to physical PVV. However, further analysis is needed to rule out the effect of time variables to determine whether these results are statistically significant.

The results of the DID model are presented in Table 3. The application of safety and security equipment was associated with a 13.9-percentage-point decrease in the percentage of physical PVV occurring in the treatment group within one year in comparison with the control group. (DID=-13.93, p < 0.05). These results are consistent with the descriptive statistics presented in Table 2. The coefficients of safety and security equipment were not significant in the DID models when psychological PVV incidence and overall PVV incidence were treated as dependent variables. The findings of this study indicate that safety and security equipment can be considered protective factors against physical PVV.

Several potential confounding factors may have affected these results. A logit model was used to estimate whether confounding factors influenced the incidence of PVV. The results are shown in Table 4. Confounding factors had no significant effect on the incidence of physical PVV, indicating that the DID models were robust. Overall, the use of safety and security equipment can significantly reduce the incidence of physical PVV.

Table 1 Characteristics of the treatment and control group over 2020 and 2021, No. (%)

Variable	Control group (N=1006)		Treatment group (N=351)		Overall (N=1357)	
	2020 (N=480)	2021 (N=526)	2020 (N=152)	2021 (N=199)	2020 (N=632)	2021 (N=715)
**Age, years**						
≤30	186(38.8)	243(46.2)	58(38.2)	70(35.2)	244(38.6)	313(43.2)
31-40	207(43.1)	199(37.8)	68(44.7)	89(44.7)	275(43.5)	288(39.7)

For peer review only - http://bmjopen.bmj.com/site/about/guidelines.xhtml

>40	87(18.1)	84(16.0)	26(17.1)	40(20.1)	113(17.9)	124(17.1)
**Gender**						
Female	461(96.0)	507(96.4)	140(92.1)	188(94.5)	601(95.1)	695(95.9)
Male	19(4.0)	19(3.6)	12(7.9)	11(5.5)	31(4.9)	30 (4.1)
**Marital status**						
Never married	162(33.8)	207(39.4)	69(45.4)	74(37.2)	231(36.6)	281(38.8)
Married	311(64.8)	310(58.9)	82(53.9)	123(61.8)	393(62.2)	433(59.7)
Divorced/widowed	7(1.5)	9(1.7)	1(0.7)	2(1.0)	8(1.3)	11(1.5)
**Job position**						
Junior / Senior nurse	313(65.2)	360(68.4)	107(70.4)	117(58.8)	420（67.9）	477（65.8）
Supervisor nurse	160(33.3)	162(30.8)	44(28.9)	78(39.2)	204（32.3）	240（33.1）
Co-chief nurse	4(0.8)	4(0.8)	1(0.7)	4(2.0)	5（0.8）	8（1.1）
Chief nurse	3(0.6)	0(0.0)	0(0.0)	0(0.0)	3（0.5）	0（0.0）
**Work experience**						
<1	19(4.0)	26(4.9)	11(7.2)	10(5.0)	30（4.7）	36（5.0）
1-5	128(26.7)	145(27.6)	52(34.2)	61(30.7)	180（28.5）	206（28.4）
6-10	156(32.5)	158(30.0)	36(23.7)	41(20.6)	192（30.4）	199（27.4）
11-15	97(20.2)	122(23.2)	29(19.1)	43(21.6)	126（19.9）	165（22.8）
16-20	40(8.3)	39(7.4)	5(3.3)	11(5.5)	45（7.1）	50（6.9）
Over 20	40(8.3)	36(6.8)	19(12.5)	33(16.6)	59（9.3）	69（9.5）
**Work in shifts**						
No	143(29.8)	144(27.4)	51(33.6)	69(34.7)	194（30.7）	438（29.4）
Yes	337(70.2)	382(72.6)	101(66.4)	130(65.3)	213（69.3）	512（70.6）
**Work between 6PM and 7AM**						
No	211(44.0)	227(43.2)	95(62.5)	133(66.8)	306（48.4）	360（49.7）
Yes	269(56.0)	299(56.8)	57(37.5)	66(33.2)	326（51.6）	365（50.3）
**Interact with patients/clients**						
No	49(10.2)	47(8.1)	39(25.7)	113(20.6)	88（13.9）	88（12.1）
Yes	431(89.8)	479(91.9)	41(74.3)	158(79.4)	544（86.1）	637（87.9）
**Number of staff present in the same work setting**						
0	5(1.0)	4(0.8)	2(1.3)	4(2.0)	7(1.1)	8(1.1)
1-5	159(33.1)	212(40.3)	64(42.1)	73(36.7)	223(35.3)	285(39.3)
6-10	181(37.7)	183(34.8)	40(26.3)	63(31.7)	221(35.0)	246(33.9)
11-15	58(12.1)	57(10.8)	26(17.1)	22(11.1)	84(13.3)	79(10.9)
Over 15	77(16.0)	70(13.3)	20(13.2)	37(18.6)	97(15.3)	107(14.8)

Note: No significant difference in baseline characteristics of nurses' personal and workplace were observed between treatment and compare groups.

Table 2 The proportion of exposure to PVV in treatment and control groups over 2020 and 2021

Type of violence	Year	Control group (N=1006)	Treatment group (N=351)	χ^2	*p*-value
Physical PVV No. (%)	2020	3 (0.6)	21 (13.8)	**54.98**	**0.000**
	2021	14 (2.7)	4 (2.0)	0.25	0.791
χ^2 (2021-2020)		**6.27**	**18.16**		
p-value		**0.014**	**0.000**		
Psychological PVV No. (%)	2020	147 (30.6)	54 (35.5)	1.28	0.272
	2021	121 (23.0)	57 (28.6)	2.48	0.122
χ^2 (2021-2020)		**7.46**	1.89		
p-value		**0.007**	0.203		
Overall PVV No. (%)	2020	147 (30.6)	58 (38.2)	2.99	0.091
	2021	124 (23.6)	57 (28.6)	1.98	0.178
χ^2 (2021-2020)		**6.34**	3.54		
p-value		**0.013**	0.067		

PVV: patients and visitors violence

Table 3 Result from the DID analysis on the use of safety and security equipment

variable	Physical PVV	Psychological PVV	Overall PVV
safety and security equipment (Std. error)	**-13.93*** **(4.893)**	6.23 (9.074)	0.88 (11.11)
Constant (Std. error)	6.73** (0.950)	27.67** (2.961)	29.44** (3.182)
Observations	18	18	18
R-squared	0.355	0.035	0.004

PVV: patients and visitors violence

*** p<0.01, * p<0.05*

Table 4 Logit regression results for control variables

Variable	Coef.	Std. Err.	*z*	*p*	95%CI	
Age	0.245	0.221	1.11	0.267	-0.187	0.677
Marital status	0.089	0.109	0.81	0.420	-0.126	0.302
Job position	0.996	0.793	1.25	0.209	-0.559	2.550
Work experience	-0.356	0.280	-1.27	0.203	-0.905	0.192
Work in shift	0.371	0.331	1.12	0.262	-0.277	1.019

For peer review only - http://bmjopen.bmj.com/site/about/guidelines.xhtml

Work between 6PM and 7AM	-0.279	0.199	-1.40	0.162	-0.670	0.111
Interact with patients/clients	-0.104	0.146	-0.71	0.476	-0.389	0.182
Numbers of staff in the same work setting	-0.137	0.190	-0.72	0.471	-0.509	0.235

Discussion

In this study we used a DID design to assess the effects of safety and security equipment on decreasing PVV among nurses. This study has several important findings. First, we found that the incidence of physical PVV in nurses significantly decreased in academic years from 2020 (pre-treatment) to 2021 (post-treatment). This demonstrates that the implementation of safety and security equipment was associated with reductions in physical PVV at the one year follow up in China, supporting the public health rationale for the use of safety and security equipment to control the spread of physical PVV, especially during the period of COVID-19. [26] We focused on patients and their family members as sources of violence because they are involved in the main types of violent incidents. [27] Given the disproportionate physical and psychological PVV against nurses, the reduction of risk through safety and security equipment, both at the individual and organizational levels, may have provided a particularly salient sense of relief only for physical PVV.

the Occurrence of PVV in the Epidemic

Our findings reveal a lower prevalence of physical PVV (1.77% in 2020 and 1.33% in 2021) and a higher prevalence of psychological PVV (14.81% in 2020 and 13.12% in 2021) attacks on nurses related to COVID-19. These results are consistent with a previous study conducted during the pandemic, which reported similar rates of PVV among nurses in China (15.8% in psychological PVV and 8.4% in physical PVV).[28] However, it is worth noting that the prevalence of PVV found in the current study appears to be lower than that observed in other country. [29] In addition, we did not find differing effects of safety and security equipment on psychological PVV between nurses in the treatment group and those in the comparison group in two years, indicating that safety and security equipment is unlikely to provide early warning when someone is speaking or acting aggressively but without any dangerous tool from entering a hospital and would only have a modest effect. [30] A recent Cochrane review stated that there is no evidence that most organizational and environmental plans reduce or minimize psychological PVV. [31] For this situation, human resource management, ongoing training, and increased communication may be helpful. [32]

Effect of the Safety and Security Equipment on Physical PVV

In this study, we made a unique contribution by quantifying the treatment effects of safety and security equipment on the incidence of PVV in public hospitals. Our findings provide evidence that implementing safety and security equipment in these hospitals led to a significant reduction of 13.9 percentage points in the incidence of physical PVV. These findings align with previous research on

security strategies that effectively reduce WPV. [33,34] Existing evidence suggests that several risk factors contribute to nurses experiencing PVV, including unsafe and crowded work environments, easy public access to the facility, and lack of security measures. [35] Safety and security equipment play a crucial role in identifying potential threats and ensuring self-protection. One key reason is their deterrent effect. The security measures implemented in hospitals, such as the use of metal detectors at entrances, restricting the carrying of knives and other dangerous materials inside medical institutions protect the personal safety of nurses. Installing functioning surveillance cameras and advanced access control systems in high-risk areas like emergency departments and waiting rooms, creating a sense of risk and discourage potential perpetrators from engaging in violent behavior and making fewer areas accessible to the public, all contribute to the personal safety of nurses. [36] In addition, installing safety and security equipment helps create a secure environment in the workplace, promoting a culture of safety and respect. When nurses feel protected and confident in their surroundings, the likelihood of PVV decreases. Security measures send a clear message that the organization prioritizes the well-being of its employees and has taken steps to ensure their safety. This fosters a positive work atmosphere, reducing tensions and conflicts that could escalate into violence.

However, the increase in physical PVV in the control group, where security measures were not implemented, raises concerns about the safety of nurses and staff members in those department. The absence of security measures could potentially expose them to a higher risk of violence. It is crucial to consider additional factors that may contribute to the varying outcomes among groups, which means the presence of specific risk factors in inpatient department. This may be attributed to the ward's environment and patient population, such as insufficient human resources, patient demographics, the acuity of illness, overcrowding, and staffing levels can all impact the occurrence of PVV. [37]

Safety and Security Equipment in Public Hospitals

Notably, the implementation of safety and security equipment rose sharply in 2022 in public hospitals in China. Most tertiary hospitals in Beijing have established safety and security equipment by installing security cameras, biometric identification, smart cards, and alarm buttons, providing security staff with walkie-talkies, and increasing the numbers of metal detectors and security channels. Perhaps because of the revision of The Law of the People's Republic of China on the Promotion of Basic Medical Care and Health. The joint efforts of the Chinese government, society, and individuals have promoted legislation that protects healthcare workers and the development of authoritative WPV guidelines. [38] The Regulations on Hospital Safety Order Management of Beijing was put into effect in July 2020, stipulating that "hospitals shall establish a safety inspection system and conduct safety inspection at the entrance of hospitals or key areas as needed to strictly prevent prohibited and restricted items from entering hospitals".[39]

The results emphasize the importance of considering the unique characteristics and requirements of different departments when formulating security strategies. The control group and treatment group may face different work environments and potential risks, necessitating tailored security measures. The outpatient and emergency departments are often high-traffic areas with a significant influx of patients

For peer review only - http://bmjopen.bmj.com/site/about/guidelines.xhtml

and visitors. Implementing security measures can minimize crowding and reduce the risk of conflicts or acts of violence. [40] Inpatient wards necessitate a distinct security approach due to the potential prolonged presence of violence from patients and their families. Security measures should focus on controlling access to these areas and ensuring the safety of both patients and healthcare professionals. Implementing access control systems with visitor management protocols, surveillance cameras in corridors, and panic buttons for staff members can help prevent unauthorized access and provide a rapid response in case of emergencies. Support and administrative areas may not directly involve patient care, they still require security measures to safeguard the well-being of healthcare professionals. Implementing access control systems, CCTV cameras, and staff identification protocols can help create a secure environment in these areas. [36] In addition, our outcomes from a panel data of departments will not just impact the incidence of PVV on the part of the perpetrators but will also raises awareness of violence prevention and control among administrators at the managerial level from the long-term, prompting them to put in place more violence-prevention measures and contributing to overall hospital improvement.

Policy Implications

In summary, the implementation of safety and security equipment in treatment group led to a significant reduction in physical PVV incidents. This highlights the effectiveness of these measures in creating a safer environment for nurses. However, the increase in PVV in the control group without safety and security equipment underscores the importance of extending security protocols to all areas of the hospital. A lot of work remains to be done to promote safety and security equipment and uptake in China, specifically, more effective legislation and policies are required from the perspective of the organization and environment to reduce the occurrence of PVV in hospitals. It is crucial for hospital administrators to carefully assess the unique needs of each department and implement comprehensive security measures to ensure the safety and well-being of healthcare staff throughout the entire facility. [41]

Limitations

This study has some limitations. First, it was limited to nurses working in the healthcare sector. We acknowledge that violence may also have been experienced by other HWs. Due to the time and resource limitations of this study, we chose only one tertiary hospital, which limits our ability to generalize the findings to all nurses in Beijing. Second, we collected data over the last two years and focused on the principal short-term impact of the current conditions; long-term effects need to be further monitored and evaluated. While we provided evidence over a relatively short time, our results may represent a starting point for the mid- and long-term effects of safety and security equipment use on nurse physical PVV decline at the time of COVID-19, when manpower is relatively scarce. Third, given that the present study included participants from various departments during the COVID-19 pandemic, the use of electronic questionnaires was preferred as a convenient and safe method of data collection for researchers and participants alike, although there may be information bias. Fourth,

For peer review only - http://bmjopen.bmj.com/site/about/guidelines.xhtml

while the sampling methods used in the first- and second-year surveys were similar, the respondents differed, making it impossible to know if the incidence of PVV changed in specific individuals. Finally, the influence of reporting biases that are likely present in the self-reported data, therefore, there may have been a recall bias in the results.

Conclusions

Our study demonstrated that organizational and environmental improvement approaches that install safety and security equipment are effective ways to decrease physical PVV in China. These findings suggest that tertiary hospitals and other health care institutions can improve nurses' safety by incorporating safety and security equipment into their environmental improvement designs. We hope that these measures can be achieved to enact a "zero" tolerance policy, and that patient-physician trust can be rebuilt in China in the coming decade. Maintenance of a sound and safe order of medical services helps to form a harmonious doctor-patient relationship.

Author affiliations
[1]Department of Nursing, Beijing Jishuitan Hospital, Capital Medical University, Beijing, China
[2]Institute of Medical Information, Chinese Academy of Medical Sciences and Peking Union Medical College, Beijing, China

Contributors JH organized, designed and conducted the study since 2021, Y-zH, DZ and J-hX conducted the survey in 2022. Y-zH organized the database, conducted the literature search, and wrote the first draft of the manuscript. Y-zH, J-hX and CZ performed the statistical analysis. JH modified the manuscript. All authors made significant contributions to the manuscript, read and approved the final version.

Funding This research was funded by the National Natural Science Foundation of China (Project Identification Code: 71804192). The funder had no influence on study design, data collection and analysis, report writing, or publication of the article.

Competing interest The authors declare that the research was conducted in the absence of any commercial or financial relationships that could be construed as a potential conflict of interest.

Patient and public involvement Patients and/or the public were not involved in the design, or conduct, or reporting, or dissemination plans of this research.

Particitence consent for publication Not required.

Ethics statement This study was approved by Ethical Review Committee of Chinese Academy of

For peer review only - http://bmjopen.bmj.com/site/about/guidelines.xhtml

Medical Sciences (IMICAMS/8/22/HREC). Written informed consent was completed by all participants prior to the survey. To ensure anonymity, names, or other identifying information were not used.

Provenance and peer review Not commissioned; externally peer reviewed.

Data availability statement The raw data supporting the conclusions of this article will be made available by the authors, without undue reservation.

Acknowledgements
We are very grateful for the assistance of the workplace violence research group of Professor Ju Huang at the Institute of Medical Information, Chinese Academy of Medical Sciences and Peking Union Medical College. Finally, we would also like to acknowledge the contributions of other staff members of our hospital who participated in the study.

Open access This is an open access article distributed in accordance with the Creative Commons Attribution 4.0 Unported (CC BY 4.0) license, which permits others to copy, redistribute, remix, transform and build upon this work for any purpose, provided the original work is properly cited, a link to the licence is given, and indication of whether changes were made. See: https://creativecommons.org/ licenses/by/4.0/.

ORCID iDs
Yanzhen Hu https://orcid.org/0000-0003-4974-9664
Ju Huang http://orcid.org/0000-0002-7810-4550

References

(1) McKay D, Heisler M, Mishori R, Catton H, Kloiber O. Attacks against healthcare personnel must stop, especially as the world fights COVID-19. Lancet. (2020) 395:1743–5. doi:10.1016/S0140-6736(20)31191-0

(2) Blumenthal D., Fowler E. J., Abrams M., Collins S. R. Covid-19—implications for the health care system. The New England Journal of Medicine. N Engl J Med. 2020;383(17):1698. doi:10.1056/NEJMx200018

(3) National Institute for Occupational Safety and Health (NIOSH). Occupational violence. Fast facts: injury data and fatality data. Centers for Disease Control and Prevention. (2016). Available online at: https://www.cdc.gov/niosh/topics/violence/fastfacts.html.

(4) Beghini, V., International Labour Office. Gender, E. & Department, I., Violence and harassment in the world of work: a guide on Convention No. 190 and Recommendation No. 206, ILO. Geneva. Available online at: https://policycommons.net/artifacts/1804382/violence-and-harassment-in-the-world-of-work/2536119/ (accessed Apr 02, 2023).

(5) The State Council of the People's Republic of China. Basic Health Care, Medicine and Health Promotion Law.

Available online at: http://www.gov.cn/xinwen/2019-12/29/content_5464861.htm (accessed Dec 29, 2019).

(6) Devi S. COVID-19 exacerbates violence against health workers. Lancet Glob. Health. (2020) ;396, 658. doi:10.1016/S0140-6736(20)31858-4

(7) Byon H. D., Sagherian K., Kim Y., et al. Nurses' experience with type ii workplace violence and underreporting during the COVID-19 pandemic. Workplace Health Saf. (2021); 21650799211031233. doi:10.1177/21650799211031233

(8) Tang, Y., Dias Martins, L. M., Wang, S. B., He, Q. X., & Huang, H. G.. The impact of nurses' sense of security on turnover intention during the normalization of COVID-19 epidemic: The mediating role of work engagement. Frontiers in public health. (2022), 10, 1051895. doi:10.3389/fpubh.2022.1051895

(9) Arnetz J. E., Goetz C. M., Arnetz B. B., Arble E. (2020). Nurse reports of stressful situations during the COVID-19 pandemic: Qualitative analysis of survey responses. Int J Environ Res Public Health. (2020) ;17(21):8126. doi:10.3390/ijerph17218126

(10) Schoenfisch AL, Pompeii LA. Security Personnel Practices and Policies in U.S. Hospitals: Findings From a National Survey. Workplace Health Saf. (2016) 64(11):531-542. doi:10.1177/2165079916653971

(11) Withnall, A. Coronavirus: Why India has had to pass new law against attacks on health care workers. The Independent, (2020). Available online at: https://www.independent.co.uk/news/world/asia/coronavirus-india-doctor-nurse-attack-law-modi-health-care-workers-a9480801.html

(12) Wiskow, C. Guidelines on Workplace Violence in the Health Sector. Available online at: https://cdn.who.int/media/docs/default-source/documents/violence-against-health-workers/wv-comparisonguidelines.pdf

(13) China National Net. The Law of the People's Republic of China on the Promotion of Basic Medical Care and Health [EB/OL]. [2020-07-15] Available online at: http://www.npc.gov.cn/npc/c30834/201912/15b7b1cfda3 74666a2d4c43d1e154 57c.shtml

(14) Karlsen IL, Andersen DR, Jaspers SØ, Sønderbo Andersen LP, Aust B. Workplace's Development of Activities and Action Plans to Prevent Violence From Clients in High-Risk Sectors. J Interpers Violence. (2023) Jan;38(1-2):NP872-NP904. doi:10.1177/08862605221086633

(15) Violence: Occupational Hazards in Hospitals. National Institute for Occupational Safety and Health; (2002). Available online at: https://www.cdc.gov/niosh/docs/2002-101/default.html

(16) Blando JD, Paul C, Szklo-Coxe M. Risk factors for workplace encounters with weapons by hospital employees. Public Health Pract (Oxf). (2021). 2:100105. doi:10.1016/j.puhip.2021.100105

(17) Sun L, Zhang W, Cao A. Associations between work-related variables and workplace violence among Chinese medical staff: A comparison between physical and verbal violence. Front Public Health. (2023).10:1043023.. doi:10.3389/fpubh.2022.1043023

(18) Li Z, Ji Y, Sun X. The impact of vaccine hesitation on the intentions to get COVID-19 vaccines: The use of the health belief model and the theory of planned behavior model. Front Public Health. (2022). 10:882909. doi:10.3389/fpubh.2022.882909

(19) Wang X, Li Y. The predictive effects of foreign language enjoyment, anxiety, and boredom on general and domain-specific English achievement in online English classrooms. Front Psychol. (2022).13:1050226. doi:10.3389/fpsyg.2022.1050226.

(20) ILO/ICN/WHO/PSI Joint Programme on Workplace Violence in the Health Sector. (2002). Framework guidelines for addressing workplace violence in the health sector / Joint Programme on Workplace Violence in

For peer review only - http://bmjopen.bmj.com/site/about/guidelines.xhtml

the Health Sector. Available online at: https://apps.who.int/iris/handle/10665/42617

(21) Chen N. A Typical Investigation Study on Workplace Violence and Influencing Factors in a Secondary Grade Public Hospital—A Cross Sectional Survey. (dissertation/master's thesis). Peking Union Medical College, Beijing, China (2019).

(22) Guo YQ, Huang J, Xu NN, Ma XJ. Worker Characteristics and Measures Associated With Patient and Visitor Violence in the COVID-19 Pandemic: A Multilevel Regression Analysis From China. Front Public Health. (2022). 10:877843. doi:10.3389/fpubh.2022.877843

(23) Abadie A. Semiparametric difference-in-differences estimators. Rev Econ Stud (2005). 72: 1–19. doi:10.1111/0034-6527.00321

(24) Dimick JB, Ryan AM. Methods for evaluating changes in health care policy: the difference-in-differences approach. JAMA. (2014).312(22):2401-2402. doi:10.1001/jama.2014.16153

(25) Card D, Krueger A. Minimum wages and employment: a case study of the fast food industry in new jersey and pennsylvania. American Economic Review (1994). 84: 772–793. doi:10.3386/w4509

(26) Zhang M, Kim R. Occupational health protection for health workers during the coronavirus disease 2019 (COVID-19) pandemic: 6P-approach in China. Glob Health J. (2021) 5:215–9. doi:10.1016/j.glohj.2021.11.007

(27) Zhao S, Liu H, Ma H, et al. Coping with Workplace Violence in Healthcare Settings: Social Support and Strategies. Int J Environ Res Public Health. (2015).12(11):14429-44. doi:10.3390/ijerph121114429

(28) Xie XM, Zhao YJ, An FR, et al. Workplace violence and its association with quality of life among mental health professionals in China during the COVID-19 pandemic. J Psychiatr Res. 2021 Mar;135:289-293.

(29) Dopelt K, Davidovitch N, Stupak A, et al. Workplace Violence against Hospital Workers during the COVID-19 Pandemic in Israel: Implications for Public Health. Int J Environ Res Public Health. 2022 Apr 12;19(8):4659.

(30) Shane Escue J, Gutierrez F, Rebecca Batts J, Lumagui M, Oloan V. Implementing Effective Interventions Against Workplace Violence. Crit Care Nurs Q. 2023 Jul-Sep 01;46(3):255-270

(31) Spelten E, Thomas B, O'Meara PF, Maguire BJ, FitzGerald D, Begg SJ. Organisational interventions for preventing and minimising aggression directed towards healthcare workers by patients and patient advocates. Cochrane Database Syst Rev. (2020). 4(4):CD012662. doi:10.1002/14651858.CD012662.pub2

(32) Yi-Lu Li, Rui-Qi Li, Dan Qiu, et al. Prevalence of Workplace Physical Violence against Health Care Professionals by Patients and Visitors: A Systematic Review and Meta-Analysis. Environ Res Public Health. 2020;17(1):299. doi:10.3390/ijerph17010299

(33) Zhao S, Qu L, Liu H, Gao L, Jiao M, Liu J, et al. Coping with Workplace Violence against General Practitioners and Nurses in Heilongjiang Province, China: Social Supports and Prevention Strategies. PLoS ONE (2016). 11(6): e0157897. doi:10.1371/journal.pone.0157897

(34) Blando JD, Cramer RJ, Szklo-Coxe M. Hospital Security Programs and Policies Related to Guns and Other Weapons. J Healthc Manag. (2019). 64(3):157-166. doi:10.1097/JHM-D-18-00046

(35) Cabilan C.J., Eley R., Snoswell C.L., Johnston A.N.B. What can we do about occupational violence in emergency departments? A survey of emergency staff. J. Nurs. Manag. (2021). 30(6):1386-1395. doi:10.1111/jonm.13294

(36) Jiang Z, Wang S, Shen Z, et al. Nurses' experience of work stress related to COVID-19 regular prevention and control in China: A qualitative study. J Nurs Manag. (2022). 30(2):375-383. doi:10.1111/jonm.13528

(37) Partridge, B., & Affleck, J. Verbal abuse and physical assault in the emergency department: Rates of violence, perceptions of safety, and attitudes towards security. Australas Emerg Nurs J. (2017). 20(3):139-145.

For peer review only - http://bmjopen.bmj.com/site/about/guidelines.xhtml

doi:10.1016/j.aenj.2017.05.001

(38) Xinhua News Agency. The Regulations on Hospital Safety Order Management of Beijing. Available online at: https://news.china.com/zw/news/13000776/20200622/38387714_all.html

(39) Hu Y, Luo Q, Li R, et al. Anti-violence measures developed by ILO and WHO: Analysis of the prevalence of workplace violence and the effects of implementation in a general hospital in China. Front Public Health. (2022).10:1049832. doi:10.3389/fpubh.2022.1049832

(40) Quinn JM, Koopman JM. Violence Risk Assessment in the Emergency Department. J Emerg Nurs. 2023 May;49(3):352-359.e1.

(41) Dafny HA, Muller A. Australian nurses' suggestions for the management of violence in the workplace: 'The people who make the policy are not the people on the floor'. J Nurs Manag. (2022). 30(6):1454-1461. doi:10.1111/jonm.13378

For peer review only - http://bmjopen.bmj.com/site/about/guidelines.xhtml

Reporting checklist for quality improvement in health care.

Based on the SQUIRE guidelines.

Instructions to authors

Complete this checklist by entering the page numbers from your manuscript where readers will find each of the items listed below.

Your article may not currently address all the items on the checklist. Please modify your text to include the missing information. If you are certain that an item does not apply, please write "n/a" and provide a short explanation.

Upload your completed checklist as an extra file when you submit to a journal.

In your methods section, say that you used the SQUIREreporting guidelines, and cite them as:

Ogrinc G, Davies L, Goodman D, Batalden P, Davidoff F, Stevens D. SQUIRE 2.0 (Standards for QUality Improvement Reporting Excellence): revised publication guidelines from a detailed consensus process

		Reporting Item	Page Number
**Title**			
	#1	Indicate that the manuscript concerns an initiative to improve healthcare (broadly defined to include the quality, safety, effectiveness, patientcenteredness, timeliness, cost, efficiency,	1

For peer review only

		and equity of healthcare)	
**Abstract**			
	#02a	Provide adequate information to aid in searching and indexing	1
	#02b	Summarize all key information from various sections of the text using the abstract format of the intended publication or a structured summary such as: background, local problem, methods, interventions, results, conclusions	1
**Introduction**			
Problem description	#3	Nature and significance of the local problem	2
Available knowledge	#4	Summary of what is currently known about the problem, including relevant previous studies	2
Rationale	#5	Informal or formal frameworks, models, concepts, and / or theories used to explain the problem, any reasons or assumptions that were used to develop the intervention(s), and reasons why the intervention(s) was expected to work	2
Specific aims	#6	Purpose of the project and of this report	2
**Methods**			
Context	#7	Contextual elements considered important at the outset of introducing the intervention(s)	3
Intervention(s)	#08a	Description of the intervention(s) in sufficient detail that others could reproduce it	3

For peer review only

Intervention(s)	#08b	Specifics of the team involved in the work	3
Study of the Intervention(s)	#09a	Approach chosen for assessing the impact of the intervention(s)	4
Study of the Intervention(s)	#09b	Approach used to establish whether the observed outcomes were due to the intervention(s)	4
Measures	#10a	Measures chosen for studying processes and outcomes of the intervention(s), including rationale for choosing them, their operational definitions, and their validity and reliability	4
Measures	#10b	Description of the approach to the ongoing assessment of contextual elements that contributed to the success, failure, efficiency, and cost	4
Measures	#10c	Methods employed for assessing completeness and accuracy of data	4
Analysis	#11a	Qualitative and quantitative methods used to draw inferences from the data	4
Analysis	#11b	Methods for understanding variation within the data, including the effects of time as a variable	4
Ethical considerations	#12	Ethical aspects of implementing and studying the intervention(s) and how they were addressed, including, but not limited to, formal ethics review and potential conflict(s) of interest	4

Results

	#13a	Initial steps of the intervention(s) and their evolution over time	6

For peer review only

		(e.g., time-line diagram, flow chart, or table), including modifications made to the intervention during the project	
	#13b	Details of the process measures and outcome	6
	#13c	Contextual elements that interacted with the intervention(s)	6
	#13d	Observed associations between outcomes, interventions, and relevant contextual elements	6
	#13e	Unintended consequences such as unexpected benefits, problems, failures, or costs associated with the intervention(s).	6
	#13f	Details about missing data	n/a
**Discussion**			
Summary	#14a	Key findings, including relevance to the rationale and specific aims	9
Summary	#14b	Particular strengths of the project	9
Interpretation	#15a	Nature of the association between the intervention(s) and the outcomes	9-10
Interpretation	#15b	Comparison of results with findings from other publications	9-10
Interpretation	#15c	Impact of the project on people and systems	9-11
Interpretation	#15d	Reasons for any differences between observed and anticipated outcomes, including the influence of context	9-10
Interpretation	#15e	Costs and strategic trade-offs, including opportunity costs	n/a
Limitations	#16a	Limits to the generalizability of the work	11

For peer review only

Limitations	#16b	Factors that might have limited internal validity such as confounding, bias, or imprecision in the design, methods, measurement, or analysis	11
Limitations	#16c	Efforts made to minimize and adjust for limitations	11
Conclusion	#17a	Usefulness of the work	9-10
Conclusion	#17b	Sustainability	9-10
Conclusion	#17c	Potential for spread to other contexts	11
Conclusion	#17d	Implications for practice and for further study in the field	11
Conclusion	#17e	Suggested next steps	11
**Other information**			
Funding	#18	Sources of funding that supported this work. Role, if any, of the funding organization in the design, implementation, interpretation, and reporting	12

None The SQUIRE 2.0 checklist is distributed under the terms of the Creative Commons Attribution License CC BY-NC 4.0. This checklist can be completed online using https://www.goodreports.org/, a tool made by the EQUATOR Network in collaboration with Penelope.ai

For peer review only

BMJ Open

Effect of Safety and Security Equipment on Patient and Visitor Violence toward Nurses in multiple public hospitals of China during COVID-19 pandemic：A retrospective, difference-in-difference analysis

Journal:	*BMJ Open*
Manuscript ID	bmjopen-2023-078598.R1
Article Type:	Original research
Date Submitted by the Author:	05-Dec-2023
Complete List of Authors:	Hu, Yanzhen; Beijing Jishuitan Hospital,<br>Huang, Ju; Chinese Academy of Medical Sciences and Peking Union Medical College, Institute of Medical Information<br>Zhao, Dan; Beijing Jishuitan Hospital, Department of Nursing<br>Zhang, Cheng; Chinese Academy of Medical Sciences & Peking Union Medical College Institute of Medical Information<br>Xia, Jinghua; Beijing Jishuitan Hospital, Department of Nursing<br>Lu, Xue-mei; Jishuitan Hospital and Fourth Medical College of Peking University, Department of Nursing
**Primary Subject Heading**:	Health policy
Secondary Subject Heading:	Health services research, Health informatics
Keywords:	Nurses, COVID-19, China, Health & safety < HEALTH SERVICES ADMINISTRATION & MANAGEMENT

SCHOLARONE™
Manuscripts

BMJ

I, the Submitting Author has the right to grant and does grant on behalf of all authors of the Work (as defined in the below author licence), an exclusive licence and/or a non-exclusive licence for contributions from authors who are: i) UK Crown employees; ii) where BMJ has agreed a CC-BY licence shall apply, and/or iii) in accordance with the terms applicable for US Federal Government officers or employees acting as part of their official duties; on a worldwide, perpetual, irrevocable, royalty-free basis to BMJ Publishing Group Ltd ("BMJ") its licensees and where the relevant Journal is co-owned by BMJ to the co-owners of the Journal, to publish the Work in this journal and any other BMJ products and to exploit all rights, as set out in our licence.

The Submitting Author accepts and understands that any supply made under these terms is made by BMJ to the Submitting Author unless you are acting as an employee on behalf of your employer or a postgraduate student of an affiliated institution which is paying any applicable article publishing charge ("APC") for Open Access articles. Where the Submitting Author wishes to make the Work available on an Open Access basis (and intends to pay the relevant APC), the terms of reuse of such Open Access shall be governed by a Creative Commons licence – details of these licences and which Creative Commons licence will apply to this Work are set out in our licence referred to above.

Other than as permitted in any relevant BMJ Author's Self Archiving Policies, I confirm this Work has not been accepted for publication elsewhere, is not being considered for publication elsewhere and does not duplicate material already published. I confirm all authors consent to publication of this Work and authorise the granting of this licence.

For peer review only

Effect of Safety and Security Equipment on Patient and Visitor Violence toward Nurses in multiple public hospitals of China during COVID-19 pandemic: A retrospective, difference-in-difference analysis

Yan-zhen Hu[1#], Ju Huang[2#], Dan Zhao [1], Cheng Zhang[2], Jing-hua Xia[1], Xue-mei Lu[1*]

[1]Department of Nursing, Beijing Jishuitan Hospital, Capital Medical University, Beijing, China
[2]Institute of Medical Information, Chinese Academy of Medical Sciences and Peking Union Medical College, Beijing, China

*** Correspondence:** Xue-mei Lu
jstlxm@163.com

[#] **Yan-zhen Hu and Ju Huang are joint first authors.**

Number of Words:4755

Number of Tables: 4

Abstract

Objectives This study aimed to analyze whether safety and security equipment decreased patient and visitor violence (PVV) toward nurses in the COVID-19 period and quantify to what extent safety and security equipment affects PVV.

Design Controlled before and after study and difference-in-difference analysis.

Setting A large hospital medical group, consisting of three public tertiary teaching hospitals, namely Xinjiekou Branch, Huilongguan Branch, and Xinlongze Branch of Beijing Jishuitan Hospital, located in the west and north parts of Beijing, China.

Participants Panel of nine departments recruited using two-step sampling method, administered online surveys in 2021 and 2022. A total of 632 eligible nurses participated in the survey in 2021, 725 eligible nurses in 2022.

Measures We assessed impacts of the safety and security equipment on the PVV. The policy has been enacted in June 2020, and the corresponding measures was established after in mid-December 2020, and therefore we use a difference-in-differences design to evaluate changes in nurses' PVV incidence. Departments are classified as either department-installed or non-installed and nurses are classified based on their department.

Results Within the treatment group, the incidence of physical PVV significantly decreased from 13.8% in 2020 to 2.0% in 2021. In the control group, the incidence of physical PVV increased from 0.6% in 2020 to 2.7% in 2021. The application of the safety and security equipment decreased the incidence of physical PVV by 13.93% (95%CI: -23.52% to -4.34%). In contrast, no difference was observed between the treatment and control groups for the incidence of psychological PVV (6.23%, 95%CI: -

For peer review only - http://bmjopen.bmj.com/site/about/guidelines.xhtml

11.56% to 24.02%) and overall PVV (0.88, 95%CI: -20.90% to 22.66%).

Conclusion The safety and security equipment reduced the incidence of physical PVV toward Nurses. For hospital managers in public hospitals, longer-term strategies roadmap for PVV prevention measures are needed to create a more supportive work environment in employees.

Keywords safety and security equipment, nurses, workplace Violence, difference-in-difference, COVID-19

Strengths and limitations of this study

- This study is the first to quantitatively assess the impacts of the safety and security equipment on PVV prevention in nurses.
- The safety and security equipment of public hospitals has been improved in China during the past two years, and the impact of safe and security equipment on the patient and visitor violence can be visualized.
- The study collected data from three public hospitals with independent operation and varied geographic in a large hospital medical group.
- Using the DID model to verify causal correlations, this study empirically ascertains the influence of safety and security measures on PVV at department level and quantify its magnitude.

Introduction

Violence and harassment against health workers (HWs) have been increasing, especially as the world fights coronavirus disease 2019 (COVID-19), which has been ongoing for 3 years, putting HWs under tremendous anxiety, burnout, and stress in hospitals. [1,2] Workplace violence (WPV) is a serious threat to HWs; the Bureau of Labor Statistics notes that of 15,980 private industry workers who experienced trauma from nonfatal WPV, 69 percent worked in health care. [3] The International Labour Office (ILO) defines WPV as incidents in which staff are abused, threatened, or assaulted in their work, which is divided into physical and psychological violence. [4] The most prevalent type of WPV in healthcare is patient and visitor violence (PVV), which can lead to physical injury (e.g., beating, kicking, slapping, stabbing, shooting, pushing, biting, and pinching, among others) and psychological harm (e.g., verbal abuse, bullying/mobbing, harassment, and threats). [5]

Globally, there have been reports of frontline HWs, especially nurses, being attacked by perpetrators because of fear, panic, misplaced anger, and misinformation during the COVID-19 pandemic. [1] According to the International Committee of the Red Cross (ICRC), more than 600 cases of violence against HWs occurred in the first 6 months of the pandemic in the world, but cases have been reported are just only a tip of the iceberg. [6] Byon et al. [7] reported that 44.4% and 67.8% of nurses experienced physical and nonphysical violence, respectively, during a five-month period early in the COVID-19 pandemic in the United States. In China, many nursing staff, particularly experienced nurses, have worked on nasopharyngeal swab collection and other epidemy-related tasks, which may have resulted in nurses working long hours, insufficient rest, and constant overtime staff shortages.

For peer review only - http://bmjopen.bmj.com/site/about/guidelines.xhtml

The shortage may aggravate the PVV. [8] In addition, anxiety, work pressure, and inadequate infection prevention and control (IPC) measures could have led to PVV during the early stage of COVID-19. [9]

With the improvement of IPC measures for frontline health workers in COVID-19, there are several countries which have developed and begun to implement coordinated strategies such as a "zero tolerance zone" for the prevention of WPV in the nurses.[10,11] The most basic element to protect against violence in the workplace is to have safe working conditions; thus, environmental controls are a key element of occupational violence and aggression prevention and management programs in healthcare worldwide, consisting of an increased presence of hospital security staff and equipment. [12] In China, the government approved the Medicine and Health Promotion Law, which took effect in June 2020 and included provisions to prevent PVV against medical workers. It emphasizes that no person or organization may threaten or endanger the personal safety of medical personnel or infringe upon their personal dignity. [6] Under this policy, safety and security measures at the organizational and environmental levels were implemented for HWs by tertiary hospitals in mid-December 2020 in Beijing, including installing security cameras and alarm buttons, providing security staff with walkie-talkies, and increasing the numbers of metal detectors and security channels. [13]

The implementation of workplace hazard prevention and control measures associated with violence against HWs has been studied worldwide for years, and the measures can be divided into two types: training plans (for internal factors of PVV, perpetrator, or recipient characteristics) and organizational and environmental plans (for external factors, policies, and the workplace environment). [14] Currently, there are a few studies that have focused on external factors. Safety and security equipment as environmental prevention strategies attract little attention. Although research on the direct effects of safety and security equipment on the decrease in PVV during COVID-19 is limited, several positive research results were reported. A cross-sectional study by Henry Ford Hospital in Michigan evaluated installed metal detectors and found that they reduced violent crimes by 65% over 18 months. The metal detectors relieve the pressure on healthcare workers and decrease PVV. [15] Blando et al. [16] also found in a cross-sectional study that metal detectors can support weapon identification and removal, enhancing safety in healthcare facilities. Yet, owing to the limitations of the cross-sectional design in the existing research, the causal associations between safety and security equipment and PVV remain unknown.

Considering the scant evidence evaluating the efficacy of safety and security equipment on PVV prevention, particularly regarding nurses, we used panel data from 2020 to 2021, 2 years before and after the implementation of safety and security equipment, respectively, adopted a difference-in-differences (DID) design, to examine the effects of safety and security equipment on PVV against nurses. The PVV incidents toward nurses during COVID-19 pandemic, and its causal correlations with implementation of safety and security measures are expected to be found in the study. In addition, the DID model used in this study could provide quantitative data to existing knowledge, quantifying the reduction rate in PVV with the implementation of safety and security measures. It offers a more

For peer review only - http://bmjopen.bmj.com/site/about/guidelines.xhtml

objective data reference for the addition of new security measures in the next phase, furthering the goal of zero tolerance for physical violence. Such findings offer valuable guidance for hospitals in other developing countries confronting similar situation. Moreover, our research augments evidence-based practices by providing empirical data on the efficacy of safety equipment.

Methods

Study Design and data collection

The data was collected from a panel of departments in three hospitals located at the west and north of Beijing, within a large, public tertiary hospital group. The hospital that provides healthcare services across nationwide, with 1,600 beds, 3,509 employees and the yearly outpatient amount reaches 2.24 million. The inclusion criteria for individual participates were full-time employees with qualification certificates and a willingness to answer the survey. The exclusion criterion was being on leave for more than a month in their department. The sample size was calculated using the double population proportion formula in PASS software based on the following assumptions: the prevalence of physical PVV from a previous study as 23%. [17] 80% power to detect a significant difference of 0.05, an effect size of 0.1, and an allocation ratio of 1:3 between two groups. Finally, after accounting for a 10% non-response rate, the sample size of this study minimum yielded 517 respondents each year (120 for the treatment group and 397 for the comparison group).

A two-step sampling method was used to recruit respondents (Figure1). First, the department-level samples were selected, which covered all major types of wards in the three hospitals. The final department-level sample comprised nine departments, including outpatient department (OD), emergency department (ED), and technical services department (TSD), general medicine, general surgery, operating room, intensive care, specialized unit, and management department, and at least two wards were included for each department. Then convenience sampling was performed to collect individual-level sample from each department. The number of nurses in each selected department was determined and used as the sampling frame. The total sample size was allocated based on the sampling frame, resulting in 10-280 individual participants in each department.

According to the Medicine and Health Promotion Law, based on the epidemiological characters of PVV, the hospital administration strategically selected three departments- OD, ED, and TSD-for the implementation of safety and security measures. Therefore, the participants from departments installed the safety and security equipment in the end of 2020 were treatment groups (OD, ED, and TSD), and the participants from departments with no safety and security equipment were control groups: general medicine, general surgery, operating room, intensive care, specialized unit, and management department. The baseline data for 2020 were collected in January 2021, and we followed-up with the selected departments that previously consented to participate in the panel for data of 2021 in January 2022. Ultimately, a total of 632 eligible nurses participated in the survey 2021 to report the PVV in 2020, of whom 152 were from the treatment group and 480 were from the control group. In survey

For peer review only - http://bmjopen.bmj.com/site/about/guidelines.xhtml

2022, 725 eligible nurses participated to report the PVV in 2021, of whom 199 were from the treatment group and 526 from the control group.

All surveys were administered online by using Wenjuanxing Forms, which is the largest computer program for conducting online surveys in China to help researchers collect data without face-to-face meetings by sending online links to nurses through WeChat. According to the set rules, all items had to be completed before submission, and there were no missing values. Therefore, the Forms which has the function of checking the questionnaire automatically to ensure all questions were answered. [18,19]

Figure 1 Sample flow diagram

Measures

The post-period for this analysis is defined as 2021. While the Medicine and Health Promotion Law was signed in June 2020, implementation was not instantaneous. The safety and security equipment were installed in the hospital in December 2020 and identified by the hospital security officer. Metal detectors (SM-SG-EDD-5000, Sinomis) and security channel (ISD-SC6550D-4YH, Sinomis) were set up at entrances in the outpatient building and emergence building to screen individuals for any potentially harmful items that could jeopardize the safety of medical personnel. Security cameras (IVS3800XF36 V5, Huawei) installed at multiple corners to covering the entire building, providing constant surveillance and aiding in the identification of potential risks or incidents; Alarm buttons (NNV-30, LonBon) installed under the triage table in ED and reception in OD, allowed immediate and discreet alerting of security personnel in case of emergencies or threatening situations. About 70 security personnel, each with a walkie-talkie (MTP3150, Xinzhiheng), facilitated swift communication and coordination among them, enabling quick responses to any security issues. These measures collectively aimed to fortify the safety and security infrastructure within the hospitals, proactively deterring and addressing potential threats against the medical staff's safety and personal dignity.

The mainly outcomes were measured by the structured Chinese Version-Revised of The Workplace Violence in the Health Sector Country Case Studies Research questionnaire, including physical PVV, psychological PVV and overall PVV. The English version was developed in 2003 by the International Council of Nurses (ICN), the World Health Organization (WHO), and Public Services International (PSI). [20] This questionnaire was revised based on the context of Chinese township hospitals and has been widely used in Chinese public hospitals.[21] The International Questionnaire-C-R includes the following four categories: demographic and workplace data; physical violence within the past 12 months; psychological workplace violence (verbal abuse, bullying/mobbing, sexual harassment, and racial harassment) within the past 12 months; and perception of effective strategies for PVV prevention. The reliability and validity of the questionnaire have been tested in studies involving experts from different areas of health in China. The Cronbach's alpha was 0.828. [22]

For peer review only - http://bmjopen.bmj.com/site/about/guidelines.xhtml

Statistical Analysis

We first present descriptive statistics of our sample, differences in PVV rates were compared using Pearson's Chi-squared test. Next, to evaluated the effect of safety and security equipment on the incidence of PVV, the DID model was used to assess the changes of PVV incidence in nurses that occurred pre-treatment versus post-treatment. To obtain the significance level of the DID estimate, we adopted a regression model of the general form [23]

$$y_{it} = \alpha + \beta intervention_i + u_i + v_t + \epsilon_{it}$$

where the subscript i indicates department, t year, and y_{it} denotes the outcome variable on the incidence of PVV varying across departments and years. The $intervention_i$ is the key explanatory variable of interest, representing the impact of safety and security equipment on the incidence of PVV. u_i is the fixed effect of the control and treatment groups (1 for the department with complete safety and security equipment and 0 for the department without the equipment); v_t is the fixed effect of the pre- and post-treatment periods (1 for 2021 and 0 for 2020).

Finally, we controlled the individual-level variables, including age, marital status, job position, work experience, work in shift, work between 6PM and 7AM, interact with patients/clients and numbers of staff in the same work setting. All data analyses were conducted using Stata 16.0. The results were considered statistically significant at p-values<0.05.

Patient and public involvement

To engage nurses in the design of this study, we conducted preliminary online communications with them. Throughout the recruitment and factual stages of the study, they were given the autonomy to decide their participation. We assured them that their decisions would not in any way impact their primary job responsibilities. This approach ensured that only voluntary participants were included, honoring their autonomy. We made concerted efforts to mitigate potential burdens or discomfort by maintaining the brevity of the online questionnaire. All participants in the survey were provided written information regarding the objective, context, and voluntary nature of their involvement in the study. The anonymity of all participants and the confidentiality of their responses were ensured. This study was approved by the Ethical Review Committee of the Chinese Academy of Medical Sciences (IMICAMS/8/22/HREC).

Results

The sociological characteristics of the treatment and control groups in 2020 and 2021 are presented in Table 1. A total of 1357 nurses (632 in 2021 and 725 in 2022) were included. From 2021 to 2022, nearly 80% of participants were aged < 40 years old. More than 95% were female and nearly 60% were married. Of the respondents, the majority (67.9% in 2021 and 65.8% in 2022) of respondents were senior nurses, and nearly 70% of respondents needed to work in shifts. More than 90% of

For peer review only - http://bmjopen.bmj.com/site/about/guidelines.xhtml

respondents were female (95.1% in 2021 and 95.9% in 2022, respectively). On average, more than half of the respondents had junior titles (67.9% in 2021 and 65.8% in 2022, respectively). Regarding their work experience, more than 75% of all respondents had less than 15 years working experience. There were no statistical differences in the sociological characteristics in both the pre- and post-intervention periods between the treatment and control groups, indicating that the two groups were comparable (p>0.05).

Table 2 shows the proportions of PVV exposure in the treatment and control groups in 2020 and 2021. Within the treatment group, the incidence of physical PVV significantly decreased from 13.8% in 2020 to 2.0% in 2021 (χ^2=18.16, p<0.05) and psychological PVV decreased from 35.5% in 2020 to 28.6% in 2021 (χ^2=1.89, p>0.05). In the control group, the incidence of physical PVV increased from 0.6% in 2020 to 2.7% in 2021(χ^2=6.27, p<0.05), and psychological PVV decreased from 30.6% in 2020to 23.0% in 2021 (χ^2=7.46, p<0.05). These results support the effect of safety and security equipment on nurses' exposure to physical PVV. However, further analysis is needed to rule out the effect of time variables to determine whether these results are statistically significant.

The results of the DID model are presented in Table 3. The application of safety and security equipment was associated with a 13.9-percentage-point decrease in the percentage of physical PVV occurring in the treatment group within one year in comparison with the control group (β=-13.93, p= 0.024). These results are consistent with the descriptive statistics presented in Table 2. The coefficients of safety and security equipment were not significant in the DID models when psychological PVV incidence and overall PVV incidence were treated as dependent variables. The findings of this study indicate that safety and security equipment can be considered protective factors against physical PVV.

Several potential confounding factors may have affected these results. A logit model was used to estimate whether confounding factors influenced the incidence of PVV. The results are shown in Table 4. Confounding factors had no significant effect on the incidence of physical PVV, indicating that the DID models were robust. Overall, the use of safety and security equipment can significantly reduce the incidence of physical PVV.

Table 1 Characteristics of the treatment and control group over 2020 and 2021, No. (%)

Variable	Control group (N=1006)		Treatment group (N=351)		Overall (N=1357)	
	2020 (N=480)	2021 (N=526)	2020 (N=152)	2021 (N=199)	2020 (N=632)	2021 (N=725)
**Age, years**						
≤30	186(38.8)	243(46.2)	58(38.2)	70(35.2)	244(38.6)	313(43.2)
31-40	207(43.1)	199(37.8)	68(44.7)	89(44.7)	275(43.5)	288(39.7)
>40	87(18.1)	84(16.0)	26(17.1)	40(20.1)	113(17.9)	124(17.1)
**Gender**						

For peer review only - http://bmjopen.bmj.com/site/about/guidelines.xhtml

Female	461(96.0)	507(96.4)	140(92.1)	188(94.5)	601(95.1)	695(95.9)
Male	19(4.0)	19(3.6)	12(7.9)	11(5.5)	31(4.9)	30 (4.1)
**Marital status**						
Never married	162(33.8)	207(39.4)	69(45.4)	74(37.2)	231(36.6)	281(38.8)
Married	311(64.8)	310(58.9)	82(53.9)	123(61.8)	393(62.2)	433(59.7)
Divorced/widowed	7(1.5)	9(1.7)	1(0.7)	2(1.0)	8(1.3)	11(1.5)
**Job position**						
Junior / Senior nurse	313(65.2)	360(68.4)	107(70.4)	117(58.8)	420（67.9）	477（65.8）
Supervisor nurse	160(33.3)	162(30.8)	44(28.9)	78(39.2)	204（32.3）	240（33.1）
Co-chief nurse	4(0.8)	4(0.8)	1(0.7)	4(2.0)	5（0.8）	8（1.1）
Chief nurse	3(0.6)	0(0.0)	0(0.0)	0(0.0)	3（0.5）	0（0.0）
**Work experience**						
<1	19(4.0)	26(4.9)	11(7.2)	10(5.0)	30（4.7）	36（5.0）
1-5	128(26.7)	145(27.6)	52(34.2)	61(30.7)	180（28.5）	206（28.4）
6-10	156(32.5)	158(30.0)	36(23.7)	41(20.6)	192（30.4）	199（27.4）
11-15	97(20.2)	122(23.2)	29(19.1)	43(21.6)	126（19.9）	165（22.8）
16-20	40(8.3)	39(7.4)	5(3.3)	11(5.5)	45（7.1）	50（6.9）
Over 20	40(8.3)	36(6.8)	19(12.5)	33(16.6)	59（9.3）	69（9.5）
**Work in shifts**						
No	143(29.8)	144(27.4)	51(33.6)	69(34.7)	194（30.7）	213（29.4）
Yes	337(70.2)	382(72.6)	101(66.4)	130(65.3)	438（69.3）	512（70.6）
**Work between 6PM and 7AM**						
No	211(44.0)	227(43.2)	95(62.5)	133(66.8)	306（48.4）	360（49.7）
Yes	269(56.0)	299(56.8)	57(37.5)	66(33.2)	326（51.6）	365（50.3）
**Interact with patients/clients**						
No	49(10.2)	47(8.1)	39(25.7)	113(20.6)	88（13.9）	88（12.1）
Yes	431(89.8)	479(91.9)	41(74.3)	158(79.4)	544（86.1）	637（87.9）
**Number of staff present in the same work setting**						
0	5(1.0)	4(0.8)	2(1.3)	4(2.0)	7(1.1)	8(1.1)
1-5	159(33.1)	212(40.3)	64(42.1)	73(36.7)	223(35.3)	285(39.3)
6-10	181(37.7)	183(34.8)	40(26.3)	63(31.7)	221(35.0)	246(33.9)
11-15	58(12.1)	57(10.8)	26(17.1)	22(11.1)	84(13.3)	79(10.9)
Over 15	77(16.0)	70(13.3)	20(13.2)	37(18.6)	97(15.3)	107(14.8)

Note: No significant difference in baseline characteristics of nurses' personal and workplace were observed between treatment and compare groups.

Table 2 The proportion of exposure to PVV in treatment and control groups over 2020 and 2021

Type of violence	Year	Control group (N=1006)	Treatment group	χ^2	*p*-value

For peer review only - http://bmjopen.bmj.com/site/about/guidelines.xhtml

			(N=351)		
Physical PVV No. (%)	2020	3 (0.6)	21 (13.8)	**54.98**	**0.000**
	2021	14 (2.7)	4 (2.0)	0.25	0.791
χ^2 (2021-2020)		**6.27**	**18.16**		
p-value		**0.014**	**0.000**		
Psychological PVV No. (%)	2020	147 (30.6)	54 (35.5)	1.28	0.272
	2021	121 (23.0)	57 (28.6)	2.48	0.122
χ^2 (2021-2020)		**7.46**	1.89		
p-value		**0.007**	0.203		
Overall PVV No. (%)	2020	147 (30.6)	58 (38.2)	2.99	0.091
	2021	124 (23.6)	57 (28.6)	1.98	0.178
χ^2 (2021-2020)		**6.34**	3.54		
p-value		**0.013**	0.067		

PVV: patients and visitors violence

Table 3 Result from the DID analysis on the use of safety and security equipment

variable	Physical PVV	Psychological PVV	Overall PVV
safety and security equipment (Std. error)	**-13.93*** **(4.893)**	6.23 (9.074)	0.88 (11.11)
Constant	6.73**	27.67**	29.44**
(Std. error)	(0.950)	(2.961)	(3.182)
Observations	18	18	18
R-squared	0.355	0.035	0.004

PVV: patients and visitors violence

*** p<0.01, * p<0.05*

Table 4 Logit regression results for control variables

Variable	Coef.	Std. Err.	*z*	*p*	95%CI	
Age	0.245	0.221	1.11	0.267	-0.187	0.677
Marital status	0.089	0.109	0.81	0.420	-0.126	0.302
Job position	0.996	0.793	1.25	0.209	-0.559	2.550
Work experience	-0.356	0.280	-1.27	0.203	-0.905	0.192
Work in shift	0.371	0.331	1.12	0.262	-0.277	1.019
Work between 6PM and 7AM	-0.279	0.199	-1.40	0.162	-0.670	0.111
Interact with patients/clients	-0.104	0.146	-0.71	0.476	-0.389	0.182
Numbers of staff in the same work setting	-0.137	0.190	-0.72	0.471	-0.509	0.235

For peer review only - http://bmjopen.bmj.com/site/about/guidelines.xhtml

Discussion

In this study we used a DID design to assess the effects of safety and security equipment on PVV incidence among nurses. This study has several important findings. We found that the implementation of safety and security equipment reduced the incidence of physical PVV in nurses significantly in academic years from 2020 (pre-treatment) to 2021 (post-treatment). This demonstrates that the safety and security equipment could protect the nurses from the physical PVV at the one year follow up in China, supporting the necessary preventive and protective measures are taken for employers to minimize occupational risks to health workers, especially during the period of COVID-19. [24] Given the disproportionate physical and psychological PVV against nurses, the reduction of risk through safety and security equipment, both at the individual and organizational levels, may have provided a particularly salient sense of relief only for physical PVV.

The Occurrence of PVV in the Epidemic

Our findings reveal a lower prevalence of physical PVV (1.77% in 2020 and 1.33% in 2021) and a higher prevalence of psychological PVV (14.81% in 2020 and 13.12% in 2021) attacks on nurses related to COVID-19. These results are consistent with a previous study conducted during the pandemic, which reported similar rates of PVV among nurses in China (15.8% in psychological PVV and 8.4% in physical PVV).[25] However, it is worth noting that the prevalence of PVV found in the current study appears to be lower than that observed in other country. [26] Evidence suggests that risk factors for PVV in the COVID-19 pandemic include high workload, crowded work environment, and increased stress level brought by the patient, and visitor within healthcare settings. [27] In this context, visible safety and security measures, such as the presence of security personnel, surveillance cameras, and restricted access to certain areas, can act as deterrents to potential aggressive behavior to patient and visitor. While these measures are primarily intended to enhance physical safety by preventing violence, their presence can also contribute to a sense of security and comfort in healthcare workers. [28] These measures might have reduced the likelihood of violent incidents stemming from hazardous weapons, offering a sense of security that could deter potential aggressive actions. [29]

Effect of the Safety and Security Equipment on PVV

In this study, we made a unique contribution by quantifying the treatment effects of safety and security equipment on the incidence of PVV in public hospitals. Our findings provide evidence that implementing safety and security equipment in these hospitals led to a significant reduction of 13.9 percentage points in the incidence of physical PVV. These findings align with previous research on security strategies that effectively reduce WPV. [30,31] Existing evidence suggests that several risk factors contribute to nurses experiencing PVV, including unsafe and crowded work environments, easy public access to the facility, and lack of security measures. [32] Safety and security equipment play a crucial role in identifying potential threats and ensuring self-protection. One key reason is their deterrent effect. The security measures implemented in hospitals, such as the use of metal detectors at entrances, restricting the carrying of knives and other dangerous materials inside medical institutions

protect the personal safety of nurses. Installing functioning surveillance cameras and advanced access control systems in high-risk areas like emergency departments and waiting rooms, creating a sense of risk and discourage potential perpetrators from engaging in violent behavior and making fewer areas accessible to the public, all contribute to the personal safety of nurses. [33] In addition, installing safety and security equipment helps create a secure environment in the workplace, promoting a culture of safety and respect. When nurses feel protected and confident in their surroundings, the likelihood of PVV decreases. Security measures send a clear message that the organization prioritizes the well-being of its employees and has taken steps to ensure their safety. This fosters a positive work atmosphere, reducing tensions and conflicts that could escalate into violence.

However, the increase in physical PVV in the control group, where security measures were not implemented, raises concerns about the safety of nurses and staff members in those department. The absence of security measures could potentially expose them to a higher risk of violence. It is crucial to consider additional factors that may contribute to the varying outcomes among groups, which means the presence of specific risk factors in inpatient department. This may be attributed to the ward's environment and patient population, such as insufficient human resources, patient demographics, the acuity of illness, overcrowding, and staffing levels can all impact the occurrence of PVV. [34]

Practically, while the safety and security equipment appear effective in curbing physical PVV, it provides limited affection in preventing psychological PVV. The physical PVV could be more visibly deterred by security equipment, and be as easily controlled by physical security measures.[35] For example, the machine recognizes that there is a danger, and security personnel can expel the relevant person from the hospital. However, although recognizing psychological PVV through smart devices like facial recognition systems is possible, the absence of design, implement and monitor a workplace policy to prevent and combat psychological PVV makes it challenging to implement necessary follow-up measures. When a system detects a patient or visitor verbally abusing HWs, there is an absence of procedures to prohibit discrimination and harassment and stigma. [36] Based on this, a broader strategy should be considered, Initiatives such as establishing a management and coordination mechanism for workplace policy to prevent and combat violence, raising awareness through various initiatives, providing training, managing human resources effectively, and improving communication can be beneficial. [37]

Safety and Security Equipment in Public Hospitals

Safety and security equipment as environmental prevention strategies attracted more and more attention. Notably, the implementation of safety and security equipment rose sharply in 2022 in public hospitals in China. Most tertiary hospitals in Beijing have established safety and security equipment by installing security cameras, biometric identification, smart cards, and alarm buttons, providing security staff with walkie-talkies, and increasing the numbers of metal detectors and security channels. Perhaps because of the revision of The Law of the People's Republic of China on the Promotion of Basic Medical Care and Health. The joint efforts of the Chinese government, society, and individuals have promoted legislation that protects healthcare workers and the development of authoritative WPV guidelines. [38] The Regulations on Hospital Safety Order Management of Beijing was put into effect

in July 2020, stipulating that "hospitals shall establish a safety inspection system and conduct safety inspection at the entrance of hospitals or key areas as needed to strictly prevent prohibited and restricted items from entering hospitals". [39]

The results emphasize the importance of considering the unique characteristics and requirements of different departments when formulating security strategies. The control group and treatment group may face different work environments and potential risks, necessitating tailored security measures. The outpatient and emergency departments are often high-traffic areas with a significant influx of patients and visitors. Implementing security measures can minimize crowding and reduce the risk of conflicts or acts of violence. [40] Inpatient wards necessitate a distinct security approach due to the potential prolonged presence of violence from patients and their families. Security measures should focus on controlling access to these areas and ensuring the safety of both patients and healthcare professionals. Implementing access control systems with visitor management protocols, surveillance cameras in corridors, and panic buttons for staff members can help prevent unauthorized access and provide a rapid response in case of emergencies. Support and administrative areas may not directly involve patient care, they still require security measures to safeguard the well-being of healthcare professionals. Implementing access control systems, CCTV cameras, and staff identification protocols can help create a secure environment in these areas. [37] In addition, our outcomes from a panel data of departments will not just impact the incidence of PVV on the part of the perpetrators but will also raises awareness of violence prevention and control among administrators at the managerial level from the long-term, prompting them to put in place more violence-prevention measures and contributing to overall hospital improvement.

Policy Implications

In summary, the implementation of safety and security equipment in treatment group led to a significant reduction in physical PVV incidents. This highlights the effectiveness of these measures in creating a safer environment for nurses. However, the increase in PVV in the control group without safety and security equipment underscores the importance of extending security protocols to all areas of the hospital. A lot of work remains to be done to promote safety and security equipment and uptake in China, specifically, more effective legislation and policies are required from the perspective of the organization and environment to reduce the occurrence of PVV in hospitals. It is crucial for hospital administrators to carefully assess the unique needs of each department and implement comprehensive security measures to ensure the safety and well-being of healthcare staff throughout the entire facility. [41]

Limitations

This study has some limitations. First, this article selected nurses as research subjects, which limits our ability to generalize the findings to all HWs. However, given that the participants from various departments were all included in the study during the COVID-19 pandemic, which could also provide evidence of PVV prevention for other HWs during the epidemic period to some extent. Second, we

For peer review only - http://bmjopen.bmj.com/site/about/guidelines.xhtml

collected data over the last two years and focused on the principal short-term impact of the current conditions; long-term effects need to be further monitored and evaluated. Third, same as previous studies, the recall bias is inevitable. However, the consistent sampling methods and electronic questionnaires were used in the first- and second-year surveys making the data collection convenient and safe, which could reduce the reporting bias to some extent. Fourth, our exclusive focus is on a single hospital, yet it's essential to note that this hospital is a large medical group comprising three public tertiary teaching hospitals. Finally, the safety and security equipment may lead to spillover effects, which could potentially lead to a contamination of the control group with the intervention effects, thereby possibly underestimating the intervention effect of the safety and security equipment on PVV.

Conclusions

Our study demonstrated that organizational and environmental improvement approaches that install safety and security equipment are effective ways to decrease physical PVV in China. These findings suggest that tertiary hospitals and other health care institutions can improve nurses' safety by incorporating safety and security equipment into their environmental improvement designs. We hope that these measures can be achieved to enact a "zero" tolerance policy, and that patient-physician trust can be rebuilt in China in the coming decade. Maintenance of a sound and safe order of medical services helps to form a harmonious doctor-patient relationship.

Author affiliations
[1]Department of Nursing, Beijing Jishuitan Hospital, Capital Medical University, Beijing, China
[2]Institute of Medical Information, Chinese Academy of Medical Sciences and Peking Union Medical College, Beijing, China

Contributors JH organized, designed and conducted the study since 2021, YZH, DZ and JHX conducted the survey in 2022.YZH organized the database, conducted the literature search, and wrote the first draft of the manuscript. YZH, JHX and CZ performed the statistical analysis. JH modified the manuscript. XML conducted Critical guidance. All authors made significant contributions to the manuscript, read and approved the final version.

Funding This research was funded by the National Natural Science Foundation of China (Project Identification Code: 71804192). The funder had no influence on study design, data collection and analysis, report writing, or publication of the article.

Competing interest The authors declare that the research was conducted in the absence of any commercial or financial relationships that could be construed as a potential conflict of interest.

Patient and public involvement Patients and/or the public were not involved in the design, or conduct,

For peer review only - http://bmjopen.bmj.com/site/about/guidelines.xhtml

or reporting, or dissemination plans of this research.

Patient consent for publication Not required.

Ethics statement This study was approved by Ethical Review Committee of Chinese Academy of Medical Sciences (IMICAMS/8/22/HREC). Written informed consent was completed by all participants prior to the survey. To ensure anonymity, names, or other identifying information were not used.

Provenance and peer review Not commissioned; externally peer reviewed.

Data availability statement The raw data supporting the conclusions of this article will be made available by the authors, without undue reservation.

Acknowledgements

We are very grateful for the assistance of the workplace violence research group of Professor Ju Huang at the Institute of Medical Information, Chinese Academy of Medical Sciences and Peking Union Medical College. Finally, we would also like to acknowledge the contributions of other staff members of our hospital who participated in the study.

Open access This is an open access article distributed in accordance with the Creative Commons Attribution 4.0 Unported (CC BY 4.0) license, which permits others to copy, redistribute, remix, transform and build upon this work for any purpose, provided the original work is properly cited, a link to the licence is given, and indication of whether changes were made. See: https://creativecommons.org/ licenses/by/4.0/.

ORCID iDs

Yanzhen Hu https://orcid.org/0000-0003-4974-9664
Ju Huang http://orcid.org/0000-0002-7810-4550

References

(1) McKay D, Heisler M, Mishori R, Catton H, Kloiber O. Attacks against healthcare personnel must stop, especially as the world fights COVID-19. Lancet. (2020) 395:1743–5. doi:10.1016/S0140-6736(20)31191-0

(2) Blumenthal D., Fowler E. J., Abrams M., Collins S. R. Covid-19—implications for the health care system. The New England Journal of Medicine. N Engl J Med. 2020;383(17):1698. doi:10.1056/NEJMx200018

(3) National Institute for Occupational Safety and Health (NIOSH). Occupational violence. Fast facts: injury data and fatality data. Centers for Disease Control and Prevention. (2016). Available online at: https://www.cdc.gov/niosh/topics/violence/fastfacts.html.

(4) Beghini, V., International Labour Office. Gender, E. & Department, I., Violence and harassment in the world of work: a guide on Convention No. 190 and Recommendation No. 206, ILO. Geneva. Available online at:

https://policycommons.net/artifacts/1804382/violence-and-harassment-in-the-world-of-work/2536119/ (accessed Apr 02, 2023).

(5) The State Council of the People's Republic of China. Basic Health Care, Medicine and Health Promotion Law. Available online at: http://www.gov.cn/xinwen/2019-12/29/content_5464861.htm (accessed Dec 29, 2019).

(6) Devi S. COVID-19 exacerbates violence against health workers. Lancet Glob. Health. (2020) ;396, 658. doi:10.1016/S0140-6736(20)31858-4

(7) Byon H. D., Sagherian K., Kim Y., et al. Nurses' experience with type ii workplace violence and underreporting during the COVID-19 pandemic. Workplace Health Saf. (2021); 21650799211031233. doi:10.1177/21650799211031233

(8) Tang, Y., Dias Martins, L. M., Wang, S. B., He, Q. X., & Huang, H. G.. The impact of nurses' sense of security on turnover intention during the normalization of COVID-19 epidemic: The mediating role of work engagement. Frontiers in public health. (2022), 10, 1051895. doi:10.3389/fpubh.2022.1051895

(9) Arnetz J. E., Goetz C. M., Arnetz B. B., Arble E. (2020). Nurse reports of stressful situations during the COVID-19 pandemic: Qualitative analysis of survey responses. Int J Environ Res Public Health. (2020) ;17(21):8126. doi:10.3390/ijerph17218126

(10) Schoenfisch AL, Pompeii LA. Security Personnel Practices and Policies in U.S. Hospitals: Findings From a National Survey. Workplace Health Saf. (2016) 64(11):531-542. doi:10.1177/2165079916653971

(11) Withnall, A. Coronavirus: Why India has had to pass new law against attacks on health care workers. The Independent, (2020). Available online at: https://www.independent.co.uk/news/world/asia/coronavirus-india-doctor-nurse-attack-law-modi-health-care-workers-a9480801.html

(12) Wiskow, C. Guidelines on Workplace Violence in the Health Sector. Available online at: https://cdn.who.int/media/docs/default-source/documents/violence-against-health-workers/wv-comparisonguidelines.pdf

(13) China National Net. The Law of the People's Republic of China on the Promotion of Basic Medical Care and Health [EB/OL]. [2020-07-15] Available online at: http://www.npc.gov.cn/npc/c30834/201912/15b7b1cfda3 74666a2d4c43d1e154 57c.shtml

(14) Karlsen IL, Andersen DR, Jaspers SØ, Sønderbo Andersen LP, Aust B. Workplace's Development of Activities and Action Plans to Prevent Violence From Clients in High-Risk Sectors. J Interpers Violence. (2023) Jan;38(1-2):NP872-NP904. doi:10.1177/08862605221086633

(15) Violence: Occupational Hazards in Hospitals. National Institute for Occupational Safety and Health; (2002). Available online at: https://www.cdc.gov/niosh/docs/2002-101/default.html

(16) Blando JD, Paul C, Szklo-Coxe M. Risk factors for workplace encounters with weapons by hospital employees. Public Health Pract (Oxf). (2021). 2:100105. doi:10.1016/j.puhip.2021.100105

(17) Sun L, Zhang W, Cao A. Associations between work-related variables and workplace violence among Chinese medical staff: A comparison between physical and verbal violence. Front Public Health. (2023).10:1043023.. doi:10.3389/fpubh.2022.1043023

(18) Li Z, Ji Y, Sun X. The impact of vaccine hesitation on the intentions to get COVID-19 vaccines: The use of the health belief model and the theory of planned behavior model. Front Public Health. (2022). 10:882909. doi:10.3389/fpubh.2022.882909

(19) Wang X, Li Y. The predictive effects of foreign language enjoyment, anxiety, and boredom on general and domain-specific English achievement in online English classrooms. Front Psychol. (2022).13:1050226.

For peer review only - http://bmjopen.bmj.com/site/about/guidelines.xhtml

doi:10.3389/fpsyg.2022.1050226.

(20) ILO/ICN/WHO/PSI Joint Programme on Workplace Violence in the Health Sector. (2002). Framework guidelines for addressing workplace violence in the health sector / Joint Programme on Workplace Violence in the Health Sector. Available online at: https://apps.who.int/iris/handle/10665/42617

(21) Chen N. A Typical Investigation Study on Workplace Violence and Influencing Factors in a Secondary Grade Public Hospital—A Cross Sectional Survey. (dissertation/master's thesis). Peking Union Medical College, Beijing, China (2019).

(22) Guo YQ, Huang J, Xu NN, Ma XJ. Worker Characteristics and Measures Associated With Patient and Visitor Violence in the COVID-19 Pandemic: A Multilevel Regression Analysis From China. Front Public Health. (2022). 10:877843. doi:10.3389/fpubh.2022.877843

(23) Abadie A. Semiparametric difference-in-differences estimators. Rev Econ Stud (2005). 72: 1–19. doi:10.1111/0034-6527.00321

(24) Zhang M, Kim R. Occupational health protection for health workers during the coronavirus disease 2019 (COVID-19) pandemic: 6P-approach in China. Glob Health J. (2021) 5:215–9. doi:10.1016/j.glohj.2021.11.007

(25) Xie XM, Zhao YJ, An FR, et al. Workplace violence and its association with quality of life among mental health professionals in China during the COVID-19 pandemic. J Psychiatr Res. 2021 Mar;135:289-293.

(26) Dopelt K, Davidovitch N, Stupak A, et al. Workplace Violence against Hospital Workers during the COVID-19 Pandemic in Israel: Implications for Public Health. Int J Environ Res Public Health. 2022 Apr 12;19(8):4659.

(27) Liu R, Li Y, An Y, Zhang L, An FR, Luo J, et al. Workplace violence against frontline clinicians in emergency departments during the COVID-19 pandemic. PeerJ. 2021; 9:e12459. doi: 10.7717/peerj.12459

(28) Hudak L, Schwimmer H, Warnock W, et al. Patient characteristics and perspectives of firearm safety discussions in the emergency department. West J Emerg Med 2021; 22:478–87. doi:10.5811/westjem.2021.3.49333

(29) Vilke GM, Billberry E, Bongbong DN, et al. Impact of Implementation of a New Weapons Screening at an Urban Emergency Department. J Emerg Med.2023;24:S0736-4679(23)00433-X.doi: 10.1016/j.jemermed. 2023.08.010.

(30) Zhao S, Qu L, Liu H, Gao L, Jiao M, Liu J, et al. Coping with Workplace Violence against General Practitioners and Nurses in Heilongjiang Province, China: Social Supports and Prevention Strategies. PLoS ONE (2016). 11(6): e0157897. doi:10.1371/journal.pone.0157897

(31) Blando JD, Cramer RJ, Szklo-Coxe M. Hospital Security Programs and Policies Related to Guns and Other Weapons. J Healthc Manag. (2019). 64(3):157-166. doi:10.1097/JHM-D-18-00046

(32) Cabilan C.J., Eley R., Snoswell C.L., Johnston A.N.B. What can we do about occupational violence in emergency departments? A survey of emergency staff. J. Nurs. Manag. (2021). 30(6):1386-1395. doi:10.1111/jonm.13294

(33) Jiang Z, Wang S, Shen Z, et al. Nurses' experience of work stress related to COVID-19 regular prevention and control in China: A qualitative study. J Nurs Manag. (2022). 30(2):375-383. doi:10.1111/jonm.13528

(34) Partridge, B., & Affleck, J. Verbal abuse and physical assault in the emergency department: Rates of violence, perceptions of safety, and attitudes towards security. Australas Emerg Nurs J. (2017). 20(3):139-145. doi:10.1016/j.aenj.2017.05.001

(35) Shane Escue J, Gutierrez F, Rebecca Batts J, Lumagui M, Oloan V. Implementing Effective Interventions Against Workplace Violence. Crit Care Nurs Q. 2023 Jul-Sep 01;46(3):255-270

(36) Spelten E, Thomas B, O'Meara PF, Maguire BJ, FitzGerald D, Begg SJ. Organisational interventions for preventing and minimising aggression directed towards healthcare workers by patients and patient advocates.

Cochrane Database Syst Rev. (2020). 4(4):CD012662. doi:10.1002/14651858.CD012662.pub2

(37) Yi-Lu Li, Rui-Qi Li, Dan Qiu, et al. Prevalence of Workplace Physical Violence against Health Care Professionals by Patients and Visitors: A Systematic Review and Meta-Analysis. Environ Res Public Health. 2020;17(1):299. doi:10.3390/ijerph17010299

(38) Xinhua News Agency. The Regulations on Hospital Safety Order Management of Beijing. Available online at: https://news.china.com/zw/news/13000776/20200622/38387714_all.html

(39) Hu Y, Luo Q, Li R, et al. Anti-violence measures developed by ILO and WHO: Analysis of the prevalence of workplace violence and the effects of implementation in a general hospital in China. Front Public Health. (2022).10:1049832. doi:10.3389/fpubh.2022.1049832

(40) Quinn JM, Koopman JM. Violence Risk Assessment in the Emergency Department. J Emerg Nurs. 2023 May;49(3):352-359.e1.

(41) Dafny HA, Muller A. Australian nurses' suggestions for the management of violence in the workplace: 'The people who make the policy are not the people on the floor'. J Nurs Manag. (2022). 30(6):1454-1461. doi:10.1111/jonm.13378

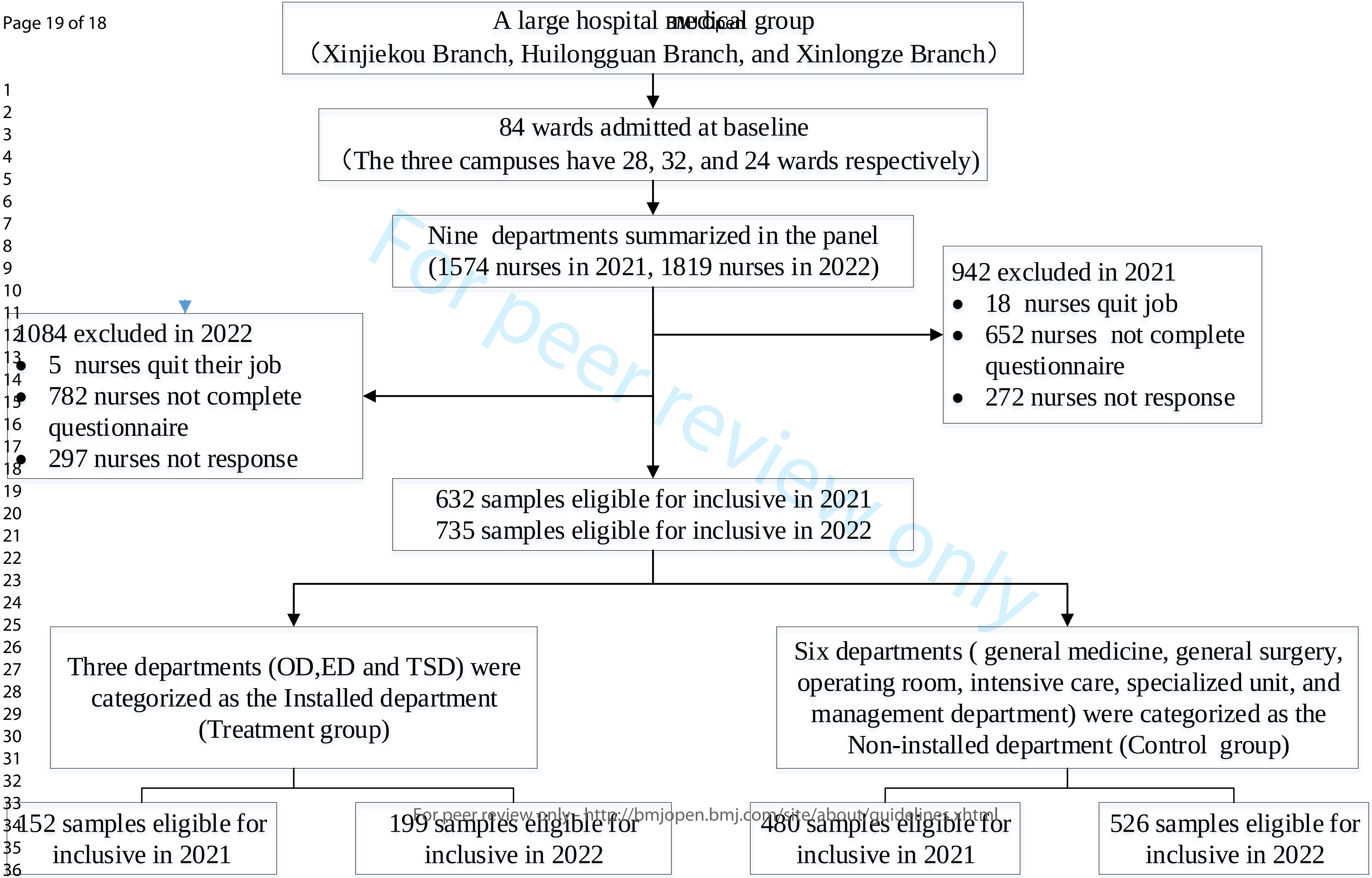

For peer review only
